# Medical Men and Matters at Vienna

**Published:** 1878-09

**Authors:** W. S. Caldwell

**Affiliations:** Vienna, Austria


### Correspondence.

### MEDICAL MEN AND MATTERS AT VIENNA.

[From our Foreign Correspondent.]

During the past winter I have been accustomed to meet in the streets of Vienna, at the same hour of the day, and nearly at the same place, two old gentlemen walking arm in arm, one of them quite short of stature, very lame, and bent with years, having nothing very remarkable in his appearance, except that his great fur coat was of a finer quality than one sees generally worn by those who go on foot in the streets of this city.

Afterwards, when I went on Friday evenings to the meetings of the "Wiener Gesellschaft der Aertze," I found this little old man occupying what appeared to be a seat of honor at a table in the centre of the room. Asking a brother physician to give me the names of the men who were seated around this table, I was greatly surprised to learn that this diminutive little man was none other than the great Skoda.

Although he never took any part in the discussions of the society, he was always present, and seemed to be an attentive listener.

Those who claim to be able to recognize qualities of mind by the size and shape of the human skull, or by the general outlines of a man's countenance, would certainly find Prof. Skoda a profound puzzle, for his appearance is anything but intellectual.

What the future may develop in the advancement of physical diagnosis we are unable to surmise, but it is not too much to say that Prof. Skoda has given this branch of medicine an impetus far exceeding anything that has been accomplished by any other person of this century, and that his name will live in all future time as one of the great lights of our profession.

He is now about 80 years of age. He resigned his professorship in 1871, retiring on his pension of $2,000 per year.

At this writing he is confined to the house by a more than usually severe attack of the gout, and fears are entertained that he will not long survive.

Prof. Bamberger, who succeeded Prof. Skoda, is about 45 years of age, wears his hair long, has a large, massive head, and looks not unlike the Henry Ward Beecher of twenty years ago. In the department of internal medicine he is perhaps the greatest man in Austria. His treatment is almost entirely expectant.

After listening to his lectures for some time, and hearing but little said of any therapeutical measures, I asked a German physician why Prof. Bamberger did not tell us something about treatment.

The reply was that physical diagnosis was his only forte; that if he encountered a case in the diagnosis of which there was doubt, his great fear was that the patient might before his death pass from under his observation, and that he might be unable to clear up the mystery by a post-mortem examination.

Prof. Duchek, although perhaps not so widely known as Prof. Bamberger, is far the better clinical teacher, and it is from him that one gets most of the practical hints in therapeutics that are to be obtained here.

In the general résumé that he gave at the end of the winter semester, of his plan of treatment, he began by laying down the following maxim:

"Give no medicines that will materially disturb the functions of life in your patient."

He said further that in the early years of his practice, he had used a wide range of remedies, and was ever ready to make trial of all the new and much-vaunted preparations that were brought forward, but having been almost invariably disappointed in their use, he had now settled down to the employment of about one dozen remedies, seldom giving any others.

Of these, the principal were quinia, digitalis, iodide and bromide of potassium, opiates, the salicylates, the acetate of ammonia, the mineral acids, ipecac, castor oil, the saline cathartics, and *occasionally* calomel. He never uses either aconite or veratrum.

He believes that the influence that these last remedies exert to lessen the muscular force of the heart, as well as the blood pressure in the arteries and veins, is, in the vast majority of cases of febrile and inflammatory disease in which they are prescribed, absolutely *injurious*.

Among the many patients I have seen treated in the Allgemeine-Krankenhaus, I have never seen a single cantharides blister used.

The poultices that one sees everywhere and in all forms in Paris, are not used here, either in the medical or surgical wards. The "Umschläge" or wet compress takes its place.

I have seen a statement made by a physician before a medical society in America, to the effect that ergot was here generally given to women in labor just at the time or a little before the child is expelled. This statement is only true *now* as far as Prof. Späth's wards are concerned, while both Carl and Gustav Braun never give ergot *before* the expulsion of the fœtus, and never give it *afterwards* unless flooding occurs, that cold externally and pressure will not control.

In fact with Carl Braun no considerable time is lost in even waiting for the effects of ergot in cases of severe post partum hœmorrhage, but the intra-uterine injection of an astringent preparation of iron is resorted to at once.

Prof. Rokitansky,* who through his researches in pathological anatomy is perhaps the most widely known of all the men connected with this school, resigned his chair in 1875, and was succeeded by Prof. Heschl, formerly of Gratz. He presided during the winter for a month, at the meetings of the "Gesellschaft der Aerzte," and often took an active part in the discussion of subjects that came up before that body. Prof. Stricker read a paper one evening on the localization of the different faculties of the mind in different sections of the brain, and, during its debate, Prof. Rokitansky gave utterance to doctrines of a materialistic

*Readers of this journal will remember that our last issue contained the news of Prof. Rokitansky's death.—Ed.

character, that would seriously have jeopardized his standing as a scientific man, and more especially as a teacher, had they been enunciated by any one occupying his position, on the other side of the Atlantic. In brief, he said that to him, it was as reasonable to suppose that every human being was possessed of an infinite mumber of immortal souls, as to suppose that he had only one. Every separate cell that the brain contained might as well be considered a small soul, as to aggregate them into one mass, and construct out of the whole one great immortal part.

He believed that the immortal part of man was the same as the immortal part of a match. When you burn the latter, carbonic acid remains. After death our bodies undergo a slower form of combustion, and the carbonic acid that is the result of this process, never is or can be destroyed. Prof. Rokitansky is a man of large frame, with a noble, intellectually-shaped head, and is apparently in robust health.

All who hold professorships in this school have the privilege of resigning at the age of 70, and drawing their full pay, $2,000 per annum, for the remainder of their lives ; and it is perhaps due to this fact that we no longer have him as a teacher.

Prof. Billroth came here from Zurich in 1867. Through his work on surgical pathology, he has achieved a world-wide fame; consequently students and physicians who resort to Vienna as a medical center, come with high hopes of what they will be able to learn from him as a teacher, but I have to see the first man who has not been disappointed in these expectations.

He is a good illustration of the error that is often committed in the selection of men to fill the chairs in the German medical schools.

Having achieved eminence by original investigation in some department of medical science, the inference is that he is qualified to teach the particular branch on which he has been able to write with such satisfaction to the profession at large; yet as a teacher, he proves, perhaps, an entire failure. The material at Prof. Billroth's command is almost unlimited; as an operator he is cool and skillful ; at his clinic you can see (if his eight assistants do not shut off your view), some of the most difficult operations in surgery, yet after all is said and done, he gives little satisfaction as an instructor of students.

Prof. Stellwag, the author of the well-known work on diseases of the eye, is a fluent lecturer, and relates an anecdote in a manner that often "brings down" the house with roars of laughter.

Though so well-known as a writer, he occupies a position as an operator and practical man, second to that of Prof. Arlt. I am told that Prof. Stellwag never achieved any considerable reputation as a practical oculist, and but for his professorship and the revenue from the sale of his book, he would, in a financial point of view, be scarcely considered successful.

Prof. Hebra, the father of modern dermatology, is about sixty-five years of age, rather short in stature, heavily built, and a genuine type of the German character. His class is composed almost exclusively of German students, the foreigners who come here preferring the shorter courses on skin diseases given by Profs. Neumann and Kaposi.

Prof. Hebra, like many other great men, though possessing a large head, has not a very tender heart. I saw him one day scrape away with a sharp spoon, the third part of a poor fellow's nose who had lupus.

As no anæsthetic was given, the man yelled most vociferously. When the doctor had finished, he asked the patient what his occupation was, and being answered that he was a shoemaker, the Prof. remarked, that that was a pity, for a man with such a magnificent voice as his, should have either been a singing master or a jackass. Prof. Hebra, as the reader is perhaps aware, does not give arsenic or other supposed specific medicines in diseases of the skin.

It is safe to say that he does not give medicines of *any kind* to one patient in twenty whom he treats for cutaneous disease, but depends entirely upon local treatment.

During the summer course we have had a successful Cæsarean section performed by Prof. Carl Braun. The subject, upon whom it was done, is a diminutive little woman, who in her childhood had suffered from rachitis, and whose pelvis in its antero-posterior diameter only measured 2¼ inches.

She had been in the hospital and her case was under observation for six weeks before the expiration of the regular term of pregnancy.

The patient was carefully watched, and when the first premonitary symptoms of labor came on, she was anæsthetized, and the operation done in the following manner :

First the abdomen was laid open in the median line from near the symphysis pubis to within three inches of the ensiform cartilage. The womb was now opened, the child and after-birth removed, great care being taken that no blood or amniotic fluid passed into the cavity of the peritoneum. The child, a girl weighing 6¾ lbs. was delivered alive.

Despite the long incision in the walls of the uterus, as soon as this organ was freed of its contents it contracted down with such force that the hæmorrhage was inconsiderable. The large chain of an écraseur was now passed down as low on the womb as could be done without bringing it to bear upon the bladder or ureters, and finally tightened so as to compress completely all the blood-vessels of that organ. The entire fundus uteri was next amputated within an inch and a half of the border of the chain.

The écraseur was left upon the stump, and brought outside of the peritoneal cavity, and the abdominal walls closed around it after the manner of the extra-abdominal management of the pedicle in cases of ovariotomy, the écraseur acting as a clamp. Two days later the stump showed some signs of bleeding, and a Spencer Wells clamp was applied over the écraseur. Four days after the operation, the patient exhibited symptoms of a partial peritonitis, which continued with a slight fever for a week longer. Excepting this slight disturbance, the patient had no bad symptoms, and made a good recovery. This, I believe, is Prof. Braun's fourth operation, but his only successful one. The removal of the entire body of the womb in connection with this operation now constitutes the exclusive mode of proceeding in cases of this kind here in Vienna.

In fact all cases that have been otherwise managed during this century have died.

Dr. Marion Sims spent some time in Vienna during the past winter, the object of his visit being, as I am informed, to collect material for a new forthcoming edition of his work on uterine surgery.

While here, the doctor was of course considerably lionized, and invited to perform gynæcological operations. Among these he did three for the removal of cancerous portions of the womb by a method called by the Germans the "*Trichterförmiche Excision*," which consists in cutting away with the scissors a funnel-shaped portion of the crevix uteri, extending, when necessary for the removal of the diseased tissue, into the body of the womb itself.

In fact, as done by Carl Braun, the procedure consists in removing the whole lower portion of the muscular tissue of the uterus, close upon the edge of the peritoneum.

Dr. Sims' patients operated upon here, all died of peritonitis within ten days of the operation. I remarked to the professor who related the facts to me, that I supposed that they selected desperate cases for Dr. Sims to operate upon. He replied that although the cases were bad ones, that did not alter the fact that they had died of the direct effects of the operation.

Dr. Bozeman spent two months here in 1876, and I find a pretty general impression among the gynæcologists of Vienna, that he is the more skilled operator of the two.

In the rivalry between these two eminent men, many here in Vienna think that Dr. Sims has carried off more than his legitimate share of the professional laurels.

American female physicians, whom we meet at every turn in Europe, have formerly been able to get private instruction in many special departments of medicine, from the docents and assistants here. This winter, unfortunately for them and for those who may come after them, we have had several of the Mary Walker type, who were not contented with any privileges short of those accorded to men, and urged their claims with such pertinacity, that the faculty were impelled to take action in the matter, which has resulted in excluding them entirely in future from everything except the lectures of Prof. Gustav Braun. His course is delivered to midwives (Hebammen), who are instructed in the management of natural labors, and emergencies that may arise before a physician can be summoned. His class this winter numbers about 70, and is mostly made up of peasant girls having a fair rudimentary education. The term of instruction varies from six to nine months. Foreign female physicians can enter this class and receive instructions with them. From 10 to 15 deliveries occur in his wards every 24 hours. His class have an opportunity to *witness* all the obstetrical operations.

The course is a very good one, but does not include everything that a lady who aspires to an eminent position in her profession would want to learn. For the benefit of the many who are in the habit of coming here, let me say that this is absolutely *all* that they can secure in Vienna.

Among the sixty or seventy American medical students and physicians who have been here attending lectures during the past year, about fifteen have been homœopathists.

We have here also a homœopathic hospital, from which has been sent out to the world some of those stunning statistics comparing the old school results of treatment at the Allgemeinen Krankenhaus with that obtained at the former institution. I have visited this hospital, and think I can explain all the difference in mortality that the friends of this exclusivism in medicine claim for it over its more aged competitor.

First, their hospital is a new one with large airy wards, and only contains fifty beds. Portions of the Allgemeinen Krankenhaus were built nearly a century ago; many of its wards have low ceilings, and it contains 3,000 beds. Without going any further, this will account for all the difference in mortality that they claim.

Then, again, they have no man on the staff of the homœopathic hospital who has any wide reputation as a surgeon, while to Billroth is sent from all Western Europe the most desperate cases for surgical treatment; cases in fact that the ordinary man dare not handle, and as a result, of course, many of them die after his operations. Among these homœopathists is one ex-president and one ex-vice-president of the Massachusetts State Homœopathic Society.

None of them ever go to this homœopathic hospital for instruction. They are much better men than the average of their school that one meets at home, and as most of them have taken special courses in physical diagnoses (in which they are generally wonderfully deficient), they are going to be difficult men to encounter, for those who come in competition with them in the future.

There has been some sickness among them during the past winter, and as far as my knowledge extends, every one of them, with a single exception, employed an old school physician.

While in Rome this spring, the ex-vice-president was taken with a severe attack of cholera morbus. He called me at 4 o'clock a. m., and I found him bathed in a cold sweat with violent cramping of the muscles of the lower extremities and discharging immensely large stools of a rice water character.

After I had examined him I said: "Doctor, there are three of our traveling companions in the next room who are homœopathic physicians, and as I do not practice that branch of medicine, you had better call one of *them.*"

He replied. "I care nothing about homœopathy *now;* I am going to die if I am not soon relieved; treat me as you would any one of your lay patients."

While selecting some instruments and other articles for a physicians' outfit in company with the ex-president, I bought a steel engraving of the medical faculty here, grouped together. I said, "Dr. C. are you not going to buy this picture also for your office?"

"Not I," he replied. "It would not look well for me to have my office decorated with the likenesses of a set of old school professors."

Yet this man will cross the Atlantic and spend months in receiving instruction from these very men whom he professes to disown, as soon as he turns his back on this city. "Consistency, thou art a jewel." W. S. Caldwell.

Vienna, Austria, July 15, 1878.

